# Medicaid Reimbursement Rates and the Quality of Mental Health Care in U.S. Nursing Homes

**DOI:** 10.1093/geroni/igaf122.3622

**Published:** 2025-12-31

**Authors:** Adrita Barooah, Pamela Nadash

**Affiliations:** University of Massachusetts Boston, Boston, Massachusetts, United States; University of Massachusetts Boston, Boston, Massachusetts, United States

## Abstract

Quality concerns about U.S. nursing homes are long-standing and have been highlighted by the COVID-19 pandemic. However, mental health quality in these settings remains understudied. Although state Medicaid reimbursement rates have been associated with overall nursing home quality, it is not known whether they affect mental health-specific outcomes. Understanding this relationship is important given facilities’ reliance on Medicaid funding and the high prevalence of psychiatric disorders among residents. Using 2019 CASPER data merged with Care Compare, Long-Term Care Focus, and the Area Health Resource File, we conducted a multilevel analysis of nursing homes nested within states to assess the association between higher Medicaid payment rates and the odds of receiving mental-health-related deficiency citations, adjusting for facility- and market-level characteristics. Results indicate that higher Medicaid reimbursement rates (Estimate = 0.0331, p = 0.0344) were significantly associated with lower odds of mental-health-related citations. Additionally, a higher proportion of Medicaid residents (p = 0.0232) and larger facility size (p = 0.0037) were associated with increased odds. Moreover, higher occupancy rates (p = 0.0053) and total staffing hours per resident day (p < 0.0001) were linked to reduced odds of deficiency citations. Study findings suggest that Medicaid payment policy, which has been associated with overall nursing home quality in prior research, may also play a crucial role in shaping mental health-related nursing home quality. The study emphasizes the importance of reexamining Medicaid reimbursement rates, given their broader implications for resident well-being, particularly for individuals with mental illness living in nursing homes.

